# Crosslinked Sulfonated Polyphenylsulfone (CSPPSU) Membranes for Elevated-Temperature PEM Water Electrolysis

**DOI:** 10.3390/membranes11110861

**Published:** 2021-11-08

**Authors:** Jedeok Kim, Akihiro Ohira

**Affiliations:** 1 Research Center for Functional Materials, Functional Clay Materials Group, National Institute for Materials Science (NIMS), 1-1 Namiki, Ibaraki, Tsukuba 305-0044, Japan; 2 Energy Storage Technology Group, Research Institute for Energy Conservation, National Institute of Advanced Industrial Science and Technology (AIST), 1-1-1 Higashi, Ibaraki, Tsukuba 305-8565, Japan; a-oohira@aist.go.jp

**Keywords:** engineering plastic PPSU, hydrocarbon electrolytes, CSPPSU membrane, PEM water electrolysis, elevated temperature

## Abstract

In order to reduce the burden on the environment, there is a need to develop non-fluorinated electrolyte membranes as alternatives to fluorinated electrolyte membranes, and water electrolysis using hydrocarbon-based electrolyte membranes has been studied in recent years. In this paper, for the first time, we report elevated-temperature water electrolysis properties of crosslinked sulfonated polyphenylsulfone (CSPPSU) membranes prepared by sulfonation and crosslinking of hydrocarbon-based PPSU engineering plastics. The sulfone groups of the CSPPSU membrane in water were stable at 85 °C (3600 h) and 150 °C (2184 h). In addition, the polymer structure of the CSPPSU membrane was stable during small-angle X-ray scattering (SAXS) measurements from room temperature to 180 °C. A current density of 456 mA/cm^2^ was obtained at 150 °C and 1.8 V in water electrolysis using the CSPPSU membrane and IrO_2_/Ti as the catalytic electrode for oxygen evolution. The stability of the CSPPSU membrane at elevated temperatures with time was evaluated. There were some issues in the assembly of the CSPPSU membrane and the catalytic electrode. However, the CSPPSU membrane has the potential to be used as an electrolyte membrane for elevated-temperature water electrolysis.

## 1. Introduction

The abnormal phenomena caused by global climate change require the construction of a social system that can provide a stable supply of energy (energy security) while reducing CO_2_ emissions to zero or below zero through carbon neutrality on a global scale. The development of renewable energy utilization technologies that do not emit CO_2_ and use sustainable energy sources, such as solar, wind, and biomass, is underway. Research on energy storage, conversion, and utilization technologies, such as batteries [1,2,3], solar cells [4], fuel cells [5], and water electrolysis [6] is being actively conducted to efficiently utilize these energies. Moreover, hydrogen, which has a high energy density, can be produced from renewable energy sources, fossil fuel reforming, industrial process by-products, biomass, water electrolysis, etc., and is suitable for energy storage systems [7,8,9].

Hydrogen production by water electrolysis has been actively researched and developed due to the effective utilization of renewable energy systems and the expansion of its use in mobile systems. There are three types of water electrolysis: alkaline water electrolysis (AWE), solid oxide water electrolysis (SOWE), and polymer electrolyte membrane water electrolysis (PEMWE). Research and development on each type of water electrolysis have been conducted to achieve larger sizes, lower cost, higher performances, and higher efficiencies [6,10,11,12,13]. For PEMWE, fluorinated electrolytes are used as the proton exchange polymer electrolyte membranes. Platinum, which is a catalyst for the hydrogen generation reaction, is used as the cathode catalyst electrode, and iridium (iridium oxide) and ruthenium (ruthenium oxide), which have excellent oxygen evolution activity, are mainly used as the anode catalyst electrode [13,14,15,16,17,18,19,20,21,22,23,24,25,26,27,28,29,30,31,32,33,34,35]. The aim is to reduce the cost and improve the performance. Fluorinated electrolytes (PFSA) [36], such as Nafion [6,12,13,15,33,37], Aquivion [6,24,33,38], and radiation-grafted ethylene tetrafluoroethylene (ETFE) membranes [39,40], have been reported to exhibit high proton conductivities, high chemical stabilities, and water electrolysis properties. Non-fluorinated hydrocarbon electrolyte membranes, such as sulfonated poly(ether ether ketone) (SPEEK) composite [41,42], polyethylene oxide grafted polyether sulfone (PES-g-PEO) [43], sulfonated polysulfone (SPSf) [44], sulfonated poly(phenylene sulfone) (sPPS) [45], and sulfonated poly(arylene ether sulfone) [46], have been reported. Hydrocarbon-based electrolyte membranes with high glass transition temperatures are being investigated as alternatives to fluorinated electrolyte membranes because they are expected to operate at elevated temperatures and have the advantages of low cost and low environmental impact due to the absence of fluorine. At the same time, higher operating temperatures (100–200 °C) are thought to be one way of increasing the efficiency of PEMWE. Moreover, the higher temperatures should improve the overvoltage of the entire cell due to the kinetic and thermodynamic advantages of the catalytic electrodes in water splitting, which improve the catalytic electrode activity [6,12], the conductivity of the electrolyte membrane [33], and the interfacial resistance between the electrolyte membrane and the catalytic electrode. Currently, water electrolysis characteristics at temperatures above 100 °C have been reported, but in those experiments, mostly fluorinated electrolyte membranes, especially Nafion membranes [33,34,35,47,48,49,50,51,52], are used. However, the application of fluorinated electrolyte membranes at temperatures above 100 °C is difficult for practical use because of the degradation of the thermal, mechanical, and chemical properties [33,53,54]. In other words, the durability of these membranes is lower at higher temperatures. There is a need to develop polymer electrolyte membranes with thermal, mechanical, and chemical stabilities at temperatures above 100 °C [5,19,55].

We have been studying the sulfonation and crosslinking of PPSU, an industrial engineering plastic, as an electrolyte membrane for fuel cells [56,57,58,59,60,61,62,63]. The conductivities and mechanical and chemical properties of the crosslinked SPPSU membranes have been found to be highly dependent on the degree of sulfonation, heat treatment, activation treatment, and additives. There is a trade-off between the improvement of the proton conductivity by highly sulfonating polymers and the improvement of physical and chemical stabilities, and we are trying to improve the performance of CSPPSU membranes to suit the application. In this study, we investigated the stabilities of the sulfone groups in water and of the polymer structure and the MEA properties of the CSPPSU membranes at elevated temperatures in elevated temperature water electrolysis.

## 2. Experimental

### 2.1. Materials

PPSU (Radel R-5000 NT; Mn = 26,000, Mw = 50,000, Mw/Mn = 1.9, glass transition temperature (Tg) = 220 °C), an engineering plastic, was provided by Solvay Specialty Polymers Japan K.K. Sulfuric acid (H_2_SO_4_, 95%), sodium hydroxide (NaOH, 97%), and sodium chloride (NaCl, 99.5%) were purchased from Nacalai Tesque, Inc, Kyoto, Japan, and dimethyl sulfoxide (DMSO, ≥99.5%) was purchased from Sigma-Aldrich Co., Ltd. MO, USA. Deionized (DI) water (15 Mohm-cm) was produced by using a Purelab Option-R 7/15 ELGA LabWater apparatus (Veolia Water Solutions & Technologies (VWS) Ltd., England, UK). Dialysis membranes (Sigma-Aldrich Co., Ltd., St. Louis, MO, USA; MWCO = 14,000) were utilized to remove excess acid from the sulfonated PPSU polymer.

### 2.2. Sulfonation of PPSU

H_2_SO_4_ (95%, 2 L) was placed in a large glass container and heated at 60 °C in an oil bath (Advantec, Tokyo, Japan; TBX243RA). Dried PPSU (70.12 g) was added to the H_2_SO_4_ solution, and sulfonation was allowed to occur at 60 °C for 2 days. After sulfonation, the product was precipitated in ice water and collected by filtration. After filtration, the product was washed until a pH of 7 using a dialysis membrane. After that, the SPPSU polymer was filtered again to remove impurities and dried by using a freeze dryer (AS ONE Corporation, Osaka, Japan; FDV-12AS).

### 2.3. Preparation of CSPPSU Membranes

A 20 wt% SPPSU solution was prepared in DMSO solvent. A glass plate (27 cm × 30 cm) was placed on a coater (KIPAE Co., Gyeonggi, Korea; KP-3000VH), and an applicator (Tester Sangyo Co., Ltd., Tokyo, Japan) was set. When the temperature of the coater was 80 °C, a 20 wt% SPPSU solution was poured onto the glass plate, and coating was carried out by moving the applicator at a speed of 4.0 mm/min. After evaporation of DMSO at 80 °C for 1 day, the SPPSU-coated glass plate was transferred to a convection oven (Yamato scientific Co., Ltd., Tokyo, Japan; DX302) for cross-linking. The SPPSU-coated glass plate was heated at 120 °C, 160 °C, and 180 °C, respectively, for 1 day each. The obtained CSPPSU membranes were about 20 cm in length, 16 cm in width and 0.07–0.130 mm in thickness.

### 2.4. Activation Treatment of CSPPSU Membranes

The CSPPSU membranes were removed from the glass plates and subjected to activation treatment to remove impurities and to improve the conduction path in the membranes. The activation involved placing the membranes in boiling water for 2 h and heating overnight at 80 °C in a 0.5 M NaOH solution. Then they were placed in boiling water for 2 h, in a 1 M H_2_SO_4_ solution at 80 °C for 2 h, and then in boiling water for 2 h. Finally, the CSPPSU membranes were dried at room temperature and used for evaluation.

### 2.5. Molecular Weight (Mw), Ion Exchange Capacity (IEC), Degree of Sulfonation (D.S.), Water Uptake (W.U.)

Gel permeation chromatography (GPC) was performed at 60 °C on a Tosoh HLC-8220 GPC equipped with a Shodex GPC LF-804 column using *N*,*N*-dimethylformamide (DMF) as the eluent. Ion exchange capacity (IEC) was defined as the millimeter equivalent of sulfonic acid groups per gram of dry sample. A portion of the membrane was soaked in 20 mL of 2 M NaCl solution and equilibrated for at least 24 h to replace the protons with sodium ions. The solution was then titrated with 0.01 M NaOH solution. The IEC value was calculated using the following equation: IEC (meq/g) = CV/W_dry_, where C (mmol/L) is the concentration of the standardized NaOH solution used in the titration (0.01 mol/L), V (L) is the volume of the standardized NaOH solution used in the titration, and W_dry_ (g) is the mass of the dry membrane. The degree of sulfonation (D.S.) of SPPSU polymer was calculated by using the following equation: D.S. (sulfonic acid groups/repeat units; R.U.) = [IEC/1000 × Fw(R.U.)]/[1 − (IEC/1000 × Fw(SO_3_))], where Fw (R.U.) = 400.45 and Fw (SO_3_) = 80.06. The water content (W.U.) of the membrane was calculated as: W.U. (%) = [(W_wet_ − W_dry_) × 100]/W_dry_. W_dry_ was obtained after placing the membrane in a dry oven at 80 °C for at least 24 h. W_wet_ was obtained after placing the dried membrane in boiling water for 1 h, immediately removing the water from the surface of the membrane, and then measuring the mass.

### 2.6. Small Angle X-ray Scattering (SAXS) Measurements of CSPPSU Membranes

The stabilities of the polymer structure of CSPPSU membranes were measured by using SAXS (beamline BL15A2 of the Photon Factory in KEK, Tsukuba, Japan [64]) and measuring the change with an increase in the temperature from room temperature to 180 °C and a decrease from 180 °C to room temperature. The X-ray beam was monochromatized to 1.2 Å. The membrane samples were placed on a temperature-controlled stage using 10002L (Linkam Scientific) to perform the measurements. The scattered photons were detected by using a two-dimensional semiconductor detector (PILATUS3 2M, W1475 × H1679 pixels, Dectris, Switzerland) at a camera distance of 170 cm, and the signal was accumulated for 30 s. The X-ray scattering images were averaged for each polar angle to obtain the radial distribution of the intensities of the scattered X-rays [62]. For SAXS measurements, the temperature was increased to 30, 80, 120, 150, and 180 °C at a rate of 10 °C/min with natural cooling from 180 to 30 °C. At each temperature, the samples were stabilized for 10–15 min before the measurements.

### 2.7. Water Stability Measurements on the CSPPSU Membranes

The stabilities of the sulfone groups of the CSPPSU membranes were evaluated by comparing the changes in their W.U., IEC, and conductivities after 3600 h (150 days) in water at 85 °C and 2184 h (91 days) in an autoclave at 150 °C. The autoclave vessel used was an HU-50 from Sanai-Kagaku (Nagoya, Japan).

### 2.8. Conductivity Measurements on the CSPPSU Membranes

**Figure 1:**
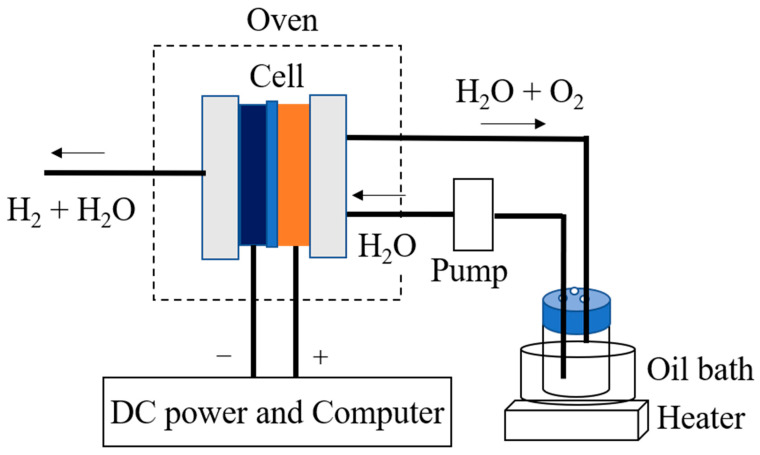
Schematic of PEM water electrolysis system.

The conductivities of the CSPPSU membranes were measured by using impedance measurements on an MTS740 membrane test system (MTS, Scribner Associates, Inc. NC, USA) in the frequency range of 1 Hz–1 MHz with a peak-to-peak voltage of 10 mV using a four-probe method. The electrode was a carbon paper electrode (area = 0.9 cm^2^) specially designed for the MTS740 system. The samples were equilibrated at the given temperatures and relative humidities (RH) for 30 min before measurement. At the same time, the conductivities of CSPPSU membranes were determined using fabricated MEAs and a water electrolysis evaluation system (Figure 1). The anode and cathode electrodes (area = 4 cm^2^) were Pt/C catalyst electrodes (EIWA co. ltd., Tokyo, Japan), which were prepared by coating 0.3 mg/cm^2^ of Pt (20 wt% Pt/C, JM) on carbon GDL (SGL 25BC, Sigracet^®^ Meitingen, Germany). The MEA was obtained by hot pressing (Model A-010D, FC-R&D company Kanagawa, Japan) for 20 min at 130 °C and 9.8 kN. A water supply unit and a pump were installed on both sides of the anode and cathode, and water was supplied to the cell at a rate of 2.0 mL/min. The electrochemical impedance spectroscopy (EIS) characteristics (AC: 10 mV, frequency: 1 Hz to 20 kHz) were evaluated at cell temperatures in the range of 80–150 °C. The water was supplied to both sides using an oil bath (EOS-200RD, AS ONE Corporation Osaka, Japan) at 80 °C without pressurization.

### 2.9. Water Electrolysis Measurements

A CSPPSU membrane was sandwiched between an IrO_2_/Ti catalytic electrode for oxygen evolution and a Pt/C catalytic electrode for hydrogen generation, and the MEA was fabricated using a hot press method to evaluate the high-temperature water electrolysis characteristics. Figure 1 shows a schematic diagram of the water electrolysis evaluation system, which we developed.

#### 2.9.1. CSPPSU Membranes for Membrane Electrode Assembly (MEA)

The size of the CSPPSU membranes for water electrolysis evaluation was 6 cm × 6 cm, and the thicknesses were in the range of 0.07–0.08 mm.

#### 2.9.2. Catalyst Electrodes for MEA

The catalytic electrode for oxygen evolution on the anode side (sample 1) was provided by Japan Carlit Co. Sample 1 had good catalytic electrode properties in the previous paper [33]. The cathode electrode for hydrogen generation was a Pt/C catalyst electrode (EIWA co. ltd. Tokyo, Japan), which was prepared by coating 0.3 mg/cm^2^ of Pt (20 wt% Pt/C, JM) on a carbon GDL (SGL 25BC, Sigracet^®^ Meitingen, Germany). The MEA was obtained by hot pressing (Model A-010D, FC-R&D company Kanagawa, Japan) at 130 °C and 9.8 kN for 20 min. The electrode areas were in the range of 4.00–4.84 cm^2^.

#### 2.9.3. Single Cell and Water Electrolysis System

The single cell consisted of an 8.8 cm × 8.8 cm Al end plate and a 6 cm × 6 cm carbon current collector with 2.2 cm × 2.2 cm channels. The evaluation system that we designed (Figure 1) consisted of a pump to supply water to the anode, an oven to control the temperature of the cells and cables, and a PC to control the electrochemical devices. The key point of the system is the use of an oven to maintain the stability of the cell temperature. The entire cell is placed in the oven, which makes the system reproducible while minimizing errors due to temperature.

#### 2.9.4. Water Electrolysis Measurements

The MEAs fabricated by hot pressing were assembled into single cells and then set into the system for evaluation. The procedures for the measurements have been described in detail in a previous paper [33].

## 3. Results and Discussions

### 3.1. Properties of the SPPSU Polymer

The yield of the synthesized SPPSU polymer was about 82%, the ion exchange capacity of the SPPSU polymer was 3.68 meq/g, and the degree of sulfonation was about 2. The weight-averaged molecular weight (Mw) of the SPPSU polymer was 134,472, the number average molecular weight (Mn) was 76,750, and Mw/Mn = 1.75. The structural properties of the SPPSU polymers determined by using ^1^H NMR spectroscopy agree with those in our previous papers [59,62].

### 3.2. Stability of the Sulfone Groups of the CSPPSU Membranes

The thermal and mechanical properties, conductivities, and fuel cell properties of the CSPPSU membranes are reported in our previous paper [62]. For water electrolysis, it is important to investigate the stabilities of the membranes under wet conditions because the membranes are always in a wet state. The stabilities of the CSPPSU membranes under wet conditions were investigated in water at 85 °C for 3600 h (150 days) and in an autoclave at 150 °C for 2184 h (91 days). Table 1 shows the IEC, W.U., and conductivity results of the CSPPSU membranes before and after the stability measurements in water. The IEC value of the CSPPSU membrane treated in water was slightly higher than that of the membrane before the water test. On the other hand, the IEC value of the CSPPSU membrane after the submersion test at 150 °C (about 0.5 MPa) was similar to that after the submersion test at 85 °C. The increase in the IEC value after submersion may be due to the cleavage of the weak part of the sulfone cross-linking in the CSPPSU polymer, resulting in an increase in the number of sulfone groups, which may have caused the increase in the W.U. and conductivity values. However, since the IEC values at 85 °C and 150 °C were similar and the appearance of the CSPPSU membrane before and after the submersion tests was the same, it is unlikely that the sulfone groups of the CSPPSU membrane degraded significantly under the test conditions.

### 3.3. Elevated-Temperature Stabilities of the CSPPSU Membranes Using SAXS

**Figure 2:**
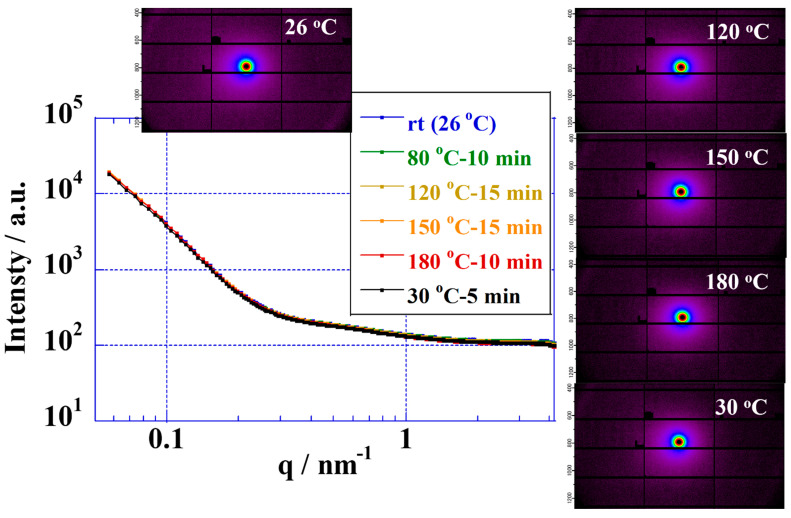
SAXS profiles of CSPPSU membranes at various temperatures.

Fluorinated electrolyte membranes are useful in elevated-temperature water electrolysis due to their high proton conductivities and their ability to form good interfaces with catalytic electrodes. However, the glass transition temperatures of fluorinated electrolyte membranes (proton form) have been reported to be in the range of 90–120 °C, and applications above the glass transition temperature have durability issues [36,65]. From stability measurements involving Nafion and Aquivion membranes at elevated temperatures performed by using SAXS, the polymer structures are unstable above 100 °C [33]. On the other hand, hydrocarbon polymers with high glass transition temperatures, such as sulfonated poly(ether ether ketone) (SPEEK) [41,42], sulfonated polysulfone (SPSf) [44], sulfonated poly(phenyl sulfone) [45,62], sulfonated poly(arylene ether sulfone) [46,58], poly(benzimidazole) [50], etc., show great promise as electrolyte membranes for water electrolysis above 100 °C [5,12,13]. The glass transition temperatures of CSPPSU membranes are about 200 °C [62], making them suitable as electrolyte membranes for water electrolysis above 100 °C. We first investigated the stabilities of CSPPSU membranes up to 180 °C using temperature-controlled SAXS (Figure 2). The polymer structures of the CSPPSU membranes did not change from room temperature to 180 °C. In addition, the polymer structures after spontaneous cooling from 180 °C to room temperature were the same as those at room temperature before the heating was applied. These results suggest that the polymer structures of the CSPPSU membranes are thermally stable up to 180 °C.

### 3.4. Conductivity Properties of the CSPPSU Membranes Using Water Electrolysis System

**Figure 3:**
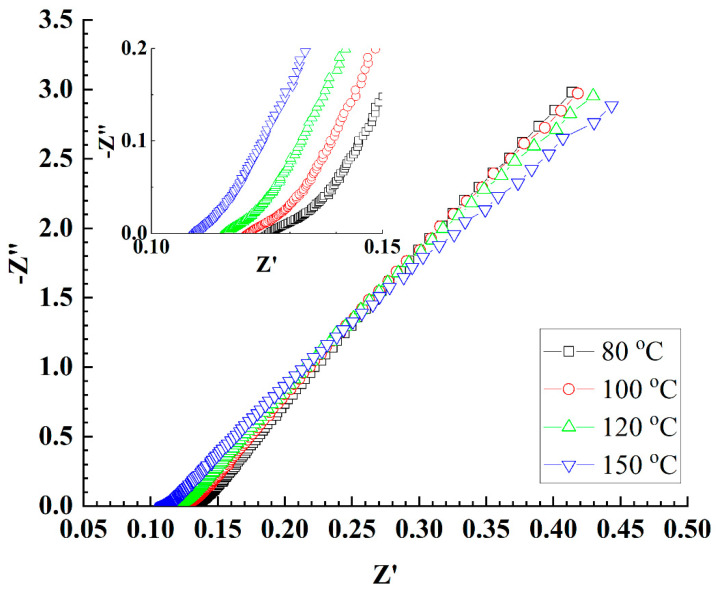
EIS properties at different cell temperatures with the water supplied at a rate of 2.0 mL/min at the temperature of 80 °C to both anode and cathode sides using PEMWE system.

MEAs were fabricated using CSPPSU membranes and Pt/C catalytic electrodes, and electrochemical impedance spectroscopy (EIS) measurements were performed using a water electrolysis system with water supplied to both sides of the anode and cathode (Figure 3). As can be seen from the inset in Figure 3, the resistances of the CSPPSU membranes decreased with an increase in the cell temperature. The conductivities of the CSPPSU membranes with these resistances were 26 mS/cm at 80 °C and 30 mS/cm at 150 °C (Table 2), which are higher than that at 80 °C and 90% RH (12 mS/cm) using the conductivity evaluation system. This may be attributed to the difference in the relative humidity. The activation energy (Ea) of the conductivities using the water electrolysis system was determined to be 0.025 eV (2.45 kJ/mol), which is smaller than the activation energy of 0.128 eV (12.41 kJ/mol) [66] for the Nafion membrane with a good conduction path. These results suggest that the conduction path of the CSPPSU membranes is saturated (percolated) above 100 °C even using this water electrolysis system without pressurization on both sides of the anode and cathode.

### 3.5. Elevated-Temperature Water Electrolysis

**Figure 4:**
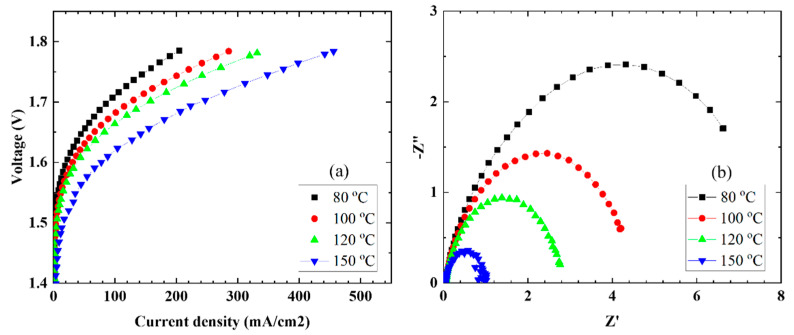
(**a**) Polarization curves and (**b**) EIS properties at different operating temperatures.

To determine the characteristics during elevated-temperature water electrolysis, the MEAs were set in single cells and evaluated at cell temperatures in the range of 80–150 °C while water was supplied at a rate of 2.0 mL/min to the anode side only. Figure 4 shows the current density–voltage (Figure 4a) and EIS (Figure 4b) characteristics as a function of cell temperature. The current densities increased with an increase in the cell temperature. A maximum current density of 456 mA/cm^2^ was obtained at a cell temperature of 150 °C and 1.8 V.

**Figure 5:**
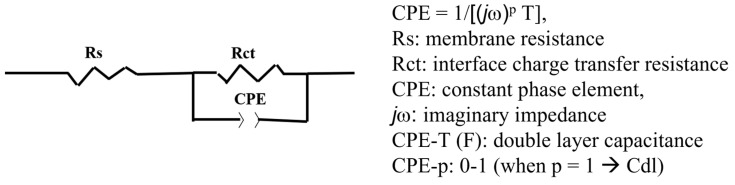
The equivalent circuit used to fit the EIS data.

From the EIS characteristics, the resistance of the entire cell tended to decrease with an increase in the cell temperature. In order to analyze the EIS characteristics, a model equivalent circuit, as shown in Figure 5, was used, assuming that the overvoltage occurred mostly on the anode side. Using the model equivalent circuit, we fitted the EIS plot in Figure 4b and calculated the resistance (Rs), interfacial charge transfer resistance (Rct), and CPE of the electrolyte membrane, and the results are summarized in Table 3. The resistance (Rs) of the CSPPSU membrane decreased when the cell temperature increased from 80 to 100 °C but did not change above 100 °C. On the other hand, Rct and CPE, which are the interfacial components between the CSPPSU membrane and the catalytic electrode, changed dramatically. As the cell temperature during water electrolysis increased, the capacitance of Rct and CPE (CPE-T) decreased. It is thought that the increase in the cell temperature increases the current density and reduces the interfacial impedance between the membrane and the catalytic electrode. On the other hand, using the same water electrolysis system and catalytic electrodes, the current density for the Nafion115 membrane was 1319 mA/cm^2^ at 150 °C and 1.8 V. The Rs, Rct, CPE-T, and CPE-p values measured using EIS were 0.024 ohm, 0.08 ohm, 0.038 F, and 0.88, respectively [33]. The performance of the CSPPSU membrane was lower than that of the Nafion membrane. The IrO_2_/Ti catalytic electrode was found to form a better interface with the fluorinated electrolyte membrane than with the CSPPSU membrane. A few studies on water electrolysis using other hydrocarbon-based electrolyte membranes have been reported [44,45,46]. Klose et al. have used all-hydrocarbon MEAs to supply water to both sides of the anode and cathode and have reported higher performances than those for Nafion membranes at a cell temperature of 80 °C [45]. However, to the best of our knowledge, ours is the first report on water electrolysis using hydrocarbon-based PEMs at temperatures above 100 °C. Elevated-temperature water electrolysis will enable high-performance production of hydrogen by improving the conductivity of the PEM, the catalytic electrode activity, and the interfacial resistance between the PEM and the catalytic electrode. We believe that research on elevated-temperature water electrolysis using hydrocarbon-based electrolyte membranes will become more active in the future.

### 3.6. Time Dependence of Elevated-Temperature Water Electrolysis

**Figure 6:**
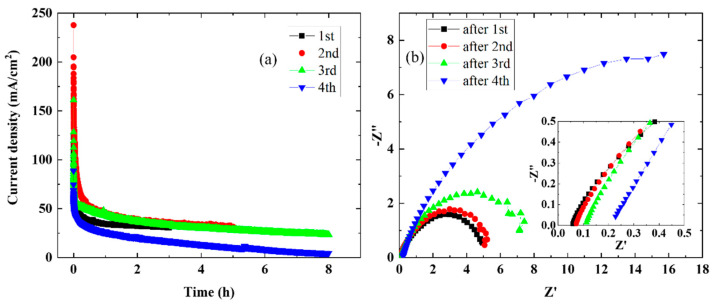
Time dependence of the single cell using sample at 120 °C and 1.7 V: (**a**) current density vs. time and (**b**) EIS properties (insert is magnified).

The stability of the MEA cell containing the CSPPSU membrane at elevated temperatures was investigated. Figure 6 shows the current density vs. time characteristics (Figure 6a) and EIS characteristics (Figure 6b) for four runs at 120 °C and 1.7 V. The large drop in current density immediately after the start of the measurement means that the cell overvoltage is large (Figure 6a). In addition, the current density decreased with each repetition of the measurement from the first to the fourth time. These results are consistent with the EIS results. The resistivity of the CSPPSU membrane and the impedance at the interface between the membrane and the catalytic electrode increased with each repetition. After the fourth measurement, the resistance of the CSPPSU membrane and the impedance at the interface between the CSPPSU membrane and the catalyst electrode were large. After evaluating the stability, the cell was disassembled, and it was found that the CSPPSU membrane and the catalytic electrode had separated. These results suggest that the increase in the impedance in the EIS characteristics is due to the separation between the membrane and the catalytic electrode. There have been reports on the use of ionomers [23], inkjet printers [28], and coating methods [29,30] to improve the interface between the electrolyte membrane and the catalytic electrode with the goal of high performance and improved durability during water electrolysis. In the future, the best method for assembling the CSPPSU membrane and the catalytic electrode to improve the current density and stability must be studied.

## 4. Conclusions

We have been studying the functionalization of PPSU, a hydrocarbon-based engineering plastic, as an alternative material to fluorinated electrolyte membranes. The functionalization of PPSU by sulfonation and crosslinking was investigated for the first time for use as an electrolyte membrane for elevated-temperature water electrolysis. The sulfone groups of the CSPPSU membrane were stable at elevated temperatures (150 °C) and high pressures and in water, and the polymer structure was thermally stable up to 180 °C as determined by using SAXS. From the elevated temperature water electrolysis using the CSPPSU membrane and IrO_2_/Ti electrode, a current density of 456 mA/cm^2^ was obtained at 150 °C and 1.8 V. The elevated-temperature stability of the CSPPSU membrane with time was evaluated. There were some issues in the assembly of the CSPPSU membrane and the catalytic electrode. On the other hand, the stabilities of the CSPPSU membranes in water and at elevated temperatures, as determined by using SAXS, suggest that these membranes can be used as electrolyte membranes for elevated-temperature water electrolysis.

## Figures and Tables

**Table 1:** Properties of the CSPPSU membranes before and after water stability tests: IEC, W.U., and conductivity.

CSPPSU Membrane	IEC (meq/g)	W.U. (%)	Conductivity (mS/cm), 80 °C	
			40% RH	90% RH
Before treatment	1.64	32.5	1.1	12.0
85 °C, 3600 h	1.72	43.5	1.3	14.0
150 °C, 2184 h	1.71	41.9	1.0	12.7

**Table 2:** Parameters obtained from EIS data (Figure 3).

	80 °C	100 °C	120 °C	150 °C
R intercept (mohm)	125	120	115	109
Conductivity (mS/cm)	26	27	28	30

**Table 3:** Parameters obtained from EIS data (Figure 4b) fitted to the equivalent circuit shown in Figure 5.

Temperature (°C)	Rs (ohm)	Rct (ohm)	CPE-T (F)	CPE-p
80	0.052	8	0.01	0.7
100	0.049	4.5	0.01	0.7
120	0.049	2.8	0.009	0.75
150	0.049	0.9	0.005	0.85
